# Exploring Mechanisms Underlying Extinction of Cue-Elicited Cocaine Seeking

**DOI:** 10.2174/157015911795017173

**Published:** 2011-03

**Authors:** Han Kong, Ming Xu

**Affiliations:** Department of Anesthesia and Critical Care, University of Chicago, Chicago, IL 60637, USA

**Keywords:** Cocaine seeking, cues, dopamine D1 receptors, c-Fos, signaling, mechanisms.

## Abstract

A prominent feature of drug addiction is that drug-associated cues can elicit drug-seeking behaviors and contribute significantly to the high propensity to relapse. We have been investigating the notion that the dopamine D1 receptor and the immediate early gene product c-Fos expressed in D1 receptor-bearing neurons mediate the development of persistent neuroadaptation in the brain dopamine system by regulating cell signaling and gene expression. We generated and analyzed genetically engineered mouse models and found that the D1 receptor and c-Fos expressed in D1 receptor-bearing neurons mediate the locomotor sensitization and reinforcing effects of cocaine. Moreover, these molecules regulate cocaine-induced dendritic remodeling, electrophysiological responses, and changes in cell signaling and gene expression in the brain. Notably, a lack of *Fos* expression in D1 receptor-bearing neurons in mice results in no change in the induction but a significantly delayed extinction of cocaine-induced conditioned place preference. These findings suggest that D1 receptor-mediated and c-Fos-regulated changes in cell signaling and gene expression may play key roles in the extinction process, and they provide a foundation for further exploring mechanisms underlying extinction of cue-elicited cocaine seeking.

## INTRODUCTION

Drug addiction is a brain disease that is characterized by the compulsive seeking and taking of drugs despite known negative consequences [1]. A key feature of drug addiction is that individuals experience intense drug craving after long abstention and are susceptible to relapse to drug seeking by exposure to drugs or drug-associated cues [1]. Memories of the drug effects or the learned association between cues and the rewarding properties of drugs are difficult to extinguish and contribute significantly to the high propensity to relapse [2]. The development of effective treatment strategies of drug addiction depends on an understanding of mechanisms underlying the development and extinction of drug-induced behaviors.

Dopamine (DA) pathways that project from the midbrain to the nucleus accumbens (NAc), amygdala (AMG), hippocampus (HIP), caudoputamen (CPu), and prefrontal cortex (PFC) play a key part in mediating the behavioral effects of drugs of abuse [3-7]. Abused drugs increase synaptic levels of DA that mediate reward and reinforcement [8, 9]. DA D1 receptors are expressed in the NAc, AMG, HIP, CPu and PFC [10]. D1 receptor agonists and antagonists can influence cocaine-induced locomotor responses, discriminative stimulus, reinforcing effects, escalated intake and reinstatement [11-17]. Using mutant mice carrying a D1 receptor gene mutation, we have demonstrated that this receptor is necessary in mediating cocaine-induced behavioral, electrophysiological, signaling and gene expression changes [18-26]. These results support the view that the D1 receptor is a key cell surface receptor in mediating the neurobiological effects of cocaine.

The persistent nature of drug addiction suggests that repeated exposure to drugs leads to stable alterations involving long-lasting changes in neuronal circuits, cell signaling and gene expression in the brain DA system [3, 4, 6, 7]. Some of these alterations are mediated by transcription factors ΔFosB and the cAMP-response element binding protein (CREB) *via* regulation of gene expression [3, 27]. The complexity of neuroadaptations and the multi-faceted nature of behavioral changes induced by repeated exposure to cocaine suggest the involvement of additional signaling molecules and their target genes [28]. The most obvious molecular response to acute stimulations by D1 receptor agonists or cocaine is a D1 receptor-dependent transient up-regulation of the immediate early gene *Fos* in the NAc, CPu, AMG and PFC [3, 20, 27]. Repeated exposure to cocaine leads to increases in *Fos* expression in the NAc [29]. c-Fos proteins form heterodimers with Jun family proteins and the resulting AP-1 transcription complexes regulate gene expression. To test the notion that c-Fos may mediate neurobiological responses to cocaine by regulating gene expression in D1 receptor-bearing neurons, we previously generated a mouse in which *Fos* is selectively mutated in this group of neurons [30]. During the course of our studies, we found that a lack of *Fos* expression in D1 receptor-bearing neurons in mice results in no change in the induction but a drastically delayed extinction of cocaine-induced conditioned place preference (CPP) [30]. These findings suggest that D1 receptor-mediated and c-Fos-regulated changes may play key roles in the extinction process, and they provide new opportunities to explore mechanisms underlying extinction of cue-elicited cocaine seeking.

## METHODS

For details of the generation of the *Fos* mutant mice and subsequent analyses, see Zhang *et al.* [30, 31].

## RESULTS

### The D1 Receptor Neuron-Specific *Fos* Mutant Mouse Model

We previously used a Cre/loxP-mediated DNA mutation strategy to engineer a mouse with a selective *Fos* mutation in D1 receptor-bearing neurons [30, 31]. We established that the acute c-Fos induction by a D1 receptor agonist SKF81297 or by cocaine was greatly reduced in the CPu and NAc of *Fos* mutant mice 7 weeks of age or older compared to that in wild-type mice [30]. Importantly, *Fos* can be significantly induced by haloperidol in both *Fos* mutant mice and wild-type. These results indicate that the *Fos* mutation is primarily limited to D1 receptor-bearing neurons in the age range we used. The *Fos* mutant mice appear healthy with no obvious abnormalities in the brain. *Fos* mutant and wild-type mice exhibit similar baseline motor activity, acute locomotor responses to SKF81297, quinpirole and haloperidol treatment, and baseline dendritic structures and dendritic spine density compared to wild-type mice [30]. Basal gene expression levels are also similar in the two groups of mice [30]. These results suggest that the *Fos* mutation did not drastically affect the development of the DA system in *Fos* mutant mice.

### c-Fos is an Intracellular Regulator of Cocaine-Induced Long-Term Changes

To investigate how c-Fos may mediate cocaine-induced persistent neurobiological changes, we previously analyzed the *Fos* mutant mouse in response to cocaine challenges using a multi-faceted approach. We found that while there were no differences in baseline activity after saline injections between *Fos* mutant mice and wild-type mice, acute cocaine administration induced significant locomotion compared to saline injections and repeated cocaine injections induced behavioral sensitization in wild-type but not in *Fos* mutant mice [30]. Whereas there was no obvious baseline difference, the number of dendrites and density of dendritic spines of neurons in the NAc shell and CPu were significantly different between the two groups of mice following repeated cocaine administration [30]. Medium spiny neurons in the NAc of mutant mice exhibited increased excitability and attenuated inhibitory responses to stimulation of D1 receptors compared to those in wild-type mice [32]. A lack of *Fos* expression in D1 receptor-bearing neurons affected FosB and ΔFosB levels and AP-1 transcription complex formation in the NAc and CPu following cocaine treatment [30]. The *Fos* mutation also affected the expression of at least two classes of target genes [30]. The first class encodes subunits of glutamate receptors including the N-methyl-D-aspartate receptor 1 subunit and the α-amino-3-hydroxy-5-methyl-4-isoxazole propionic acid receptor subunit 2. The second class encodes signaling molecules β-catenin, Cdk5 and p35. Together, these findings suggest that c-Fos is an intracellular regulator of cocaine-induced alterations in gene expression and neuronal activity, reorganization of neuronal circuits, and the development and manifestation of behavioral sensitization.

### c-Fos is Involved in Regulating the Extinction of Cue-Elicited Cocaine Seeking

Environmental cues previously associated with reinforcing drugs can play a key part in relapse to drug seeking behaviors in humans [1, 2]. This classical conditioning in which previously neutral cues acquire secondary reinforcing properties when paired with a primary reinforcer can be tested in the CPP paradigm [17, 33]. CPP is thought to model aspects of cue-elicited drug seeking behaviors because experimental animals approach and remain in contact with cues that have been paired with the effects of the drugs. CPP is thus useful in studying mechanisms related to cue-elicited drug seeking and its extinction. CPP is sensitive and can be reliably produced at low doses of drugs and with as little as one pairing of drugs. This paradigm typically produces a monophasic dose-response curve. Testing for CPP is under drug-free conditions and drug-induced effects do not influence the final behavioral outcome. For these reasons, the CPP paradigm has been increasingly used to dissect neurobiological mechanisms underlying aspects of acquisition, extinction and reinstatement of drug seeking behaviors, particularly in genetically modified mouse models [17].

We investigated whether c-Fos-mediated intracellular changes contribute to the development and extinction of cue-elicited cocaine seeking by using *Fos* mutant mice to perform a CPP study. We found that neither *Fos* mutant nor wild-type mice showed bias in freely exploring the two compartments [30]. Following four pairings each with cocaine and saline, both *Fos* mutant mice and wild-type mice developed CPP at two cocaine doses whereas saline-paired groups did not [30]. Significantly, there was no difference in the expression of CPP between the two groups of mice under any of the training conditions [30]. Wild-type mice showed CPP extinction after 10 days of training. In sharp contrast, *Fos* mutant mice still exhibited CPP after 10, 12, 14, 16 and 18 days of extinction training and they did not exhibit extinction till after 22 days of training [30]. Wild-type and *Fos* mutant mice exhibited similar cocaine-induced reinstatement of CPP [30]. These results demonstrate that a *Fos* mutation in D1 receptor-bearing neurons does not obviously affect acquisition and reinstatement but significantly delays extinction of cocaine-induced CPP, and they suggest that D1 receptor-mediated and c-Fos-regulated changes may play key roles in the extinction of cue-elicited cocaine seeking.

## DISCUSSION

Repeated exposure to cocaine induces long-lasting changes in behavior, neuronal circuits and gene expression *via* DA D1 receptors. c-Fos is a transcription factor that is robustly induced by D1 receptor agonists and cocaine in D1 receptor-bearing neurons. By generating and analyzing a mouse in which *Fos* is mostly mutated in D1 receptor-bearing neurons, we found that c-Fos is an intracellular regulator of cocaine-induced alterations in gene expression and neuronal activity, reorganization of neuronal circuits, and the development of behavioral sensitization. Moreover, our findings suggest that c-Fos-regulated mechanisms within the D1 receptor-bearing neurons may play key roles in the extinction of cue-elicited cocaine seeking.

Much has been learned about the basis of the development of cocaine-induced persistent changes. Mechanisms underlying the extinction of such long-lasting changes, however, remain much less investigated. Multiple DA signals exist during the extinction of cocaine self-administration [34]. Moreover, DA is involved in reward-related learning [35]. Our results together with these findings strongly implicate the involvement of dopaminergic mechanisms in the extinction of cocaine seeking behaviors. One of our ongoing research directions is to identify DA receptor subtypes, signaling pathways as well as target molecules that are involved in the extinction process. Cocaine can induce D1 receptor-dependent activation of ERK and CREB and expression of c-Fos and ΔFosB, and regulates PKA signaling in locomotor activation, reward and relapse behavioral paradigms [3, 19, 24, 26, 27]. Based on available evidence, we expect most if not all of these signaling molecules and transcription factors to be involved in CPP acquisition. Because extinction likely involves new learning to inhibit previous mechanisms [36], and extinction can be readily reinstated by a single cocaine injection, we expect the time course and levels of activation and expression of these molecules to be quantitatively different from the induction phase following extinction training. There might also be shifts in the pattern of activation of the signaling molecules and transcription factors following extinction training compared to the acquisition phase. Neurotrophic factors and glutamate receptors may also be involved in the extinction process [36].

The basolateral AMG (BLA) mediates both acquisition and extinction of conditioned fear, and the PFC, HIP and NAc modulate these processes [36, 37]. These same brain structures are also part of the circuitry underlying cue-elicited cocaine seeking, including acquisition and extinction [38]. The BLA mediates learning conditioned associations between the rewarding effects of cocaine and cues. The NAc modulates motivation for cocaine seeking by integrating information from the AMG and PFC and relaying it to motor output structures, and it mediates reinforcement. Our findings suggest that D1 receptor-mediated signaling through c-Fos is necessary for changes in neuronal activity and synaptic transmission that underlie the acquisition and extinction of cue-elicited cocaine seeking [30, 32]. These findings and the *Fos* mutant mouse model provide new opportunities to dissect D1 receptor-mediated and c-Fos-regulated electrophysiological changes following acquisition and especially extinction of cue-elicited cocaine seeking in the reward circuitry.

CPP is thought to reflect aspects of cue-elicited drug seeking yet this paradigm may not fully model addicted states in humans as drugs are delivered non-contingently and only a few pairings are required to produce CPP. We will consider using the self-administration paradigm which is thought to reflect active drug seeking and taking as an alternative in the future [18, 34]. We have been using this paradigm to study roles of DA receptors in the positive reinforcing effects of cocaine [18]. Another concern with the CPP paradigm is that pharmacological investigations are difficult, as it is difficult to generate graded dose-effect relationships. To overcome this, we may need to train mice with one of several cocaine doses in one context and with a reference dose in another context. Despite these unresolved issues, our findings should provide new opportunities to explore mechanisms underlying extinction of cue-elicited cocaine seeking.

## References

[1] Dackis C, O'Brien CP (2005). Neurobiology of addiction: treatment and public policy ramifications. Nat. Neurosci.

[2] O'Brien CP, Childress AR, Ehrman R, Robbins SJ (1998). Conditioning factors in drug abuse: can they explain compulsion?. J. Psychopharmacol.

[3] Hyman SE, Malenka RC, Nestler EJ (2006). Neural mechanisms of addiction: the role of reward-related learning and memory. Ann. Rev. Neurosci.

[4] Kalivas PW, O’Brien CP (2008). Drug addiction as a pathology of staged neuroplasticity. Neuropsychopharmacology.

[5] Koob GF (1992). Drugs of abuse: anatomy, pharmacology and function of reward pathways. Trends Pharmacol. Sci.

[6] Koob GF (2006). The neurobiology of addiction: a neuroadaptational view relevant for diagnosis. Addiction.

[7] Shalev U, Grimm JW, Shaham Y (2002). Neurobiology of relapse to heroin and cocaine seeking: a review. Pharmacol. Rev.

[8] Di Chiara G, Imperato A (1998). Drugs abused by humans preferentially increase synaptic dopamine concentrations in the mesolimbic system of freely moving rats. Proc. Natl. Acad. Sci. USA.

[9] Ritz MC, Lamb RJ, Goldberg SR, Kuhar MJ (1987). Cocaine receptors on dopamine transporters are related to self-administration of cocaine. Science.

[10] Missale C, Nash SR, Robinson SW, Jaber M, Caron MG (1998). Dopamine receptors: from structure to function. Physiol. Rev.

[11] Anderson SM, Bari AA, Pierce RC (2003). Administration of the D1-like dopamine receptor antagonist SCH-23390 into the medial nucleus accumbens shell attenuates cocaine priming-induced reinstatement of drug-seeking behavior in rats. Psychopharmacology.

[12] Cabib S, Castellano C, Cestari V, Filibeck U, Puglisi-Allegra S (1991). D_1_ and D_2_ receptor antagonists differently affect cocaine-induced locomotor hyperactivity in the mouse. Psychopharmacology.

[13] Caine SB, Koob GF (1994). Effects of dopamine D-1 and D-2 antagonists on cocaine-self-administration under different schedules of reinforcement in the rat. J. Pharmacol. Exp. Ther.

[14] Katz JL, Kopajtic TA, Myers KA, Mitkus RJ, Chider M (1999). Behavioral effects of cocaine: interactions with D1 dopaminergic antagonists and agonists in mice and squirrel monkeys. J. Pharmacol. Exp. Ther.

[15] Self DW, Barnhart WJ, Lehman DA, Nestler EJ (1996). Opposite modulation of cocaine-seeking behavior by D1- and D2-like dopamine receptor agonists. Science.

[16] Tella SR (1994). Differential blockade of chronic versus acute effects of intravenous cocaine by dopamine receptor antagonists. Pharmacol. Biochem. Behav.

[17] Tzschentke TM (2007). Measuring reward with the conditioned place preference (CPP) paradigm: update of the last decade. Addict. Biol.

[18] Caine SB, Gabriel KI, Berkowitz JS, Gold LH, Koob GF, Tonegawa S, Zhang J, Xu M (2007). Role of the dopamine D1 receptor in cocaine self-administration: studies with D1 receptor mutant mice. J. Neurosci.

[19] Jiao H, Zhang L, Gao F, Lou D, Zhang J, Xu M (2007). Dopamine D1 and D3 receptors oppositely regulate NMDA- and cocaine-induced MAPK signaling *via* NMDA receptor phosphorylation. J. Neurochem.

[20] Moratalla R, Xu M, Tonegawa S, Graybiel AM (1996). Cellular responses to psychomotor stimulant and neuroleptic drugs are abnormal in mice lacking the D1 dopamine receptor. Proc. Natl. Acad. Sci. USA.

[21] Xu M, Guo Y, Vorhees CV, Zhang J (2000). Behavioral responses to cocaine and amphetamine in D1 dopamine receptor mutant mice. Brain Res.

[22] Xu M, Hu XT, Cooper DC, Moratalla R, Graybiel AM, White FJ, Tonegawa S (1994). Elimination of cocaine-induced hyperactivity and dopamine-mediated neurophysiological effects in dopamine D1 receptor mutant mice. Cell.

[23] Xu M, Moratalla R, Gold LH, Hiroi N, Koob GF, Graybiel AM, Tonegawa S (1994). Dopamine D1 receptor mutant mice are deficient in striatal expression of dynorphin and in dopamine-mediated behavioral responses. Cell.

[24] Zhang D, Zhang L, Lou D, Nakabeppu Y, Zhang J, Xu M (2002). The dopamine D1 receptor is a critical mediator for cocaine-induced gene expression. J. Neurochem.

[25] Zhang D, Zhang L, Tang Y, Zhang Q, Lou D, Sharp F, Zhang J, Xu M (2005). Gene expression changes induced by repeated cocaine administration through the dopamine D1 receptors. Neuropsychopharmacology.

[26] Zhang L, Lou D, Jiao H, Zhang D, Wang X, Ying X, Zhang J, Xu M (2004). Cocaine-induced intracellular signaling and gene expression are oppositely regulated by the dopamine D1 and D3 receptors. J. Neurosci.

[27] McClung CA, Nestler EJ (2003). Regulation of gene expression and cocaine reward by CREB and DeltaFosB. Nat. Neurosci.

[28] Graybiel AM, Moratalla R, Robertson HA (1990). Amphetamine and cocaine induce drug-specific activation of the c-*fos* gene in striosome-matrix compartments and limbic subdivisions of the striatum. Proc. Natl. Acad. Sci. USA.

[29] Crombag HS, Jedynak JP, Redmond K, Robinson TE, Hope BT (2002). Locomotor sensitization to cocaine is associated with increased Fos expression in the accumbens, but not in the caudate. Behav. Brain. Res.

[30] Zhang J, Zhang L, Jiao H, Zhang Q, Zhang D, Lou D, Katz J, Xu M (2006). c-*fos* facilitates acquisition and extinction of cocaine-induced persistent change. J. Neurosci.

[31] Zhang J, Zhang D, Slane J, Behbehani M, Tsien J, Xu M (2002). c-*fos* regulates neuronal excitability and survival. Nat. Genet.

[32] Hu XT, Nasif FJ, Zhang J, Xu M (2008). *Fos* regulates neuronal activity in the nucleus accumbens. Neurosci. Lett.

[33] Bardo MT, Bevins RA (2000). Conditioned place preference: what does it add to our preclinical understanding of drug reward?. Psychopharmacology.

[34] Stuber GD, Wightman RM, Carelli RM (2005). Extinction of cocaine self-administration reveals functionally and temporally distinct dopaminergic signals in the nucleus accumbens. Neuron.

[35] Schultz W (1998). Predictive reward signal of dopamine neurons. J. Neurophysiol.

[36] Myers KM, Davis M (2007). Mechanisms of fear extinction. Mol. Psychiatry.

[37] Pezze MA, Feldon J (2004). Mesolimbic dopaminergic pathways in fear conditioning. Prog. Neurobiol.

[38] Feltenstein MW, See RE (2008). The neurocircuitry of addiction: an overview. Br. J. Pharm.

